# Engineering Dental Tissues Using Biomaterials with Piezoelectric Effect: Current Progress and Future Perspectives

**DOI:** 10.3390/jfb14010008

**Published:** 2022-12-22

**Authors:** Sumanta Ghosh, Wei Qiao, Zhengbao Yang, Santiago Orrego, Prasanna Neelakantan

**Affiliations:** 1 Faculty of Dentistry, The University of Hong Kong, Hong Kong SAR, China; 2 Department of Mechanical Engineering & Department of Materials Science and Engineering, City University of Hong Kong, Hong Kong, China; 3 Oral Health Sciences Department, Kornberg School of Dentistry, Temple University, Philadelphia, PA 19140, USA; 4 Bioengineering Department, College of Engineering, Temple University, Philadelphia, PA 19140, USA

**Keywords:** bio-piezoelectricity, dentin-pulp complex, mechanosensitive ion channel, piezo-1 receptor, piezoelectric biomaterials, regenerative endodontics, tissue engineering

## Abstract

Dental caries and traumatic injuries to teeth may cause irreversible inflammation and eventual death of the dental pulp. Nevertheless, predictably, repair and regeneration of the dentin-pulp complex remain a formidable challenge. In recent years, smart multifunctional materials with antimicrobial, anti-inflammatory, and pro-regenerative properties have emerged as promising approaches to meet this critical clinical need. As a unique class of smart materials, piezoelectric materials have an unprecedented advantage over other stimuli-responsive materials due to their inherent capability to generate electric charges, which have been shown to facilitate both antimicrobial action and tissue regeneration. Nonetheless, studies on piezoelectric biomaterials in the repair and regeneration of the dentin-pulp complex remain limited. In this review, we summarize the biomedical applications of piezoelectric biomaterials in dental applications and elucidate the underlying molecular mechanisms contributing to the biological effect of piezoelectricity. Moreover, we highlight how this state-of-the-art can be further exploited in the future for dental tissue engineering.

## 1. Introduction

The dental pulp is a vital tissue that is enclosed within the highly mineralized dentin. Injury to the dental pulp due to microbial infection (caries) and traumatic injuries to the tooth result in its inflammation and subsequent necrosis if left untreated. More than 25% of school children and 33% of adults experience trauma to their permanent teeth before 19 years of age [1]. While root canal treatment is predictable and highly successful in mature teeth, the same is not true for immature teeth because of thin and weak dentinal walls, which predispose them to fracture under stress overload [2]. Regeneration of the dentin-pulp complex is a preferred treatment for such cases, and this procedure aims to engineer metabolically active pulp or pulp-like tissue that can form new dentin (i.e., dentin-pulp complex), prevent re-infection of the tooth, and improve the functionality of the tooth [1,2]. However, this remains a clinical challenge given the lack of multifunctional materials that can control infection and simultaneously support the regeneration of tissues.

**Figure 1:**
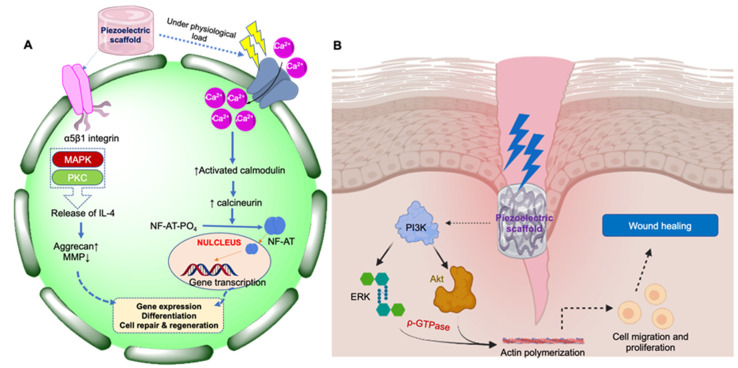
Schematic representation of cell signaling pathways through which piezoelectric materials elicit their action in tissue repair and regeneration. (**A**) Calcium/calmodulin pathway in bone, cartilage, and tendon; (**B**) PI3K-Akt-based signaling pathway for wound healing in skin.

Physiological load in tissues such as the skin, bone, cartilage, periodontal ligament, and dentin results in the activation of specific molecular cell signaling processes that trigger the generation of electrical potential, a property termed piezoelectricity. The generation of electric potential results in depolarization (or the potential difference between the cell surface and the cytoplasm), inducing various cell signaling pathways such as the calcium/calmodulin pathway in bone, cartilage, and tendon [3,4]. The resultant electric potential depolarizes the cell membrane and leads to the opening of several voltage-gated Ca^2+^ ion channels across the cell membranes. Then, a rapid influx of Ca^2+^ ions result in increased intracellular Ca^2+^ ions concentrations, which causes the dephosphorylation of nuclear factors of activated cells (NF-AT). Subsequently, the translocation of NF-AT into the nucleus regulates the transcription of several genes, which results in several cellular functions, including cell proliferation and stem cell differentiation (Figure 1). The piezoelectric potential also induces phosphatidylinositol 3-kinase (PI3K)/protein kinase B (Akt) based signaling, which regulates the fundamental aspects of physiological wound healing processes, including cell proliferation, differentiation, and migration, angiogenesis, and metabolism [4,5]. Additionally, this pathway also stimulates the extracellular signal-regulated kinase (ERK) and GTPase-mediated actin polymerization process, which ultimately leads to cutaneous wound healing by promoting keratinocyte proliferation and migration [5]. Upregulation of TGF-β, BMP, and COL-III by piezoelectric mechanisms is crucial for bone and cartilage tissue repair or regeneration [6]. 

Furthermore, to elucidate the cellular and molecular level mechanisms of the piezoelectric potential generated from the piezo materials, many gene/protein biomarkers have been identified for different tissue regeneration over the past few years. For instance, Bhang et al. [5] identified that several intra/extra-cellular proteins such as transforming growth factor (TGF-β), vascular endothelial growth factor (VEGF), collagen type-3 (Col III), integrin α5, proliferating cell nuclear antigen (PCNA), keratin 17, MMP2, CD68, and fibronectin are expressed after the induction of the piezoelectric potential on human dermal fibroblasts (HDFs), keratinocytes, and hMSCs, which directly indicate accelerated inflammation modulation, re-epithelialization, proliferation, granulation, and remodeling stages of the wound healing process. Additionally, Vignesh et al. reported the upregulation of anti-runt-related transcription factor 2 (RUNX2), osteocalcin (OCN), and osteopontin (OPN) gene expression, which enhances alkaline phosphatase and calcium deposition, indicating the osteogenetic differentiation ability of piezoelectric biomaterials for bone regeneration [3,7,8]. Nevertheless, it has also been reported that the piezoelectric potential positively influences the upregulation of several neurotropic factors such as NGF, GDNF, and BDNF, which are required for axonal regeneration and neuronal differentiation for nerve tissue engineering [9,10]. Thus, different genes or biomarkers involved in the different physiological activities of the piezoelectric potential are summarized in Table 1.

Predictable regeneration of the dentin-pulp complex requires a material to be endowed with multiple properties, including antimicrobial, immune modulatory, and cell differentiation to several phenotypes (odontogenic, vascular, and neural) [11,12,13,14]. Given that biological tissues such as dentin respond to mechanical stimulus by producing piezoelectric potential, there is a remarkable interest in developing mechano-responsive, piezoelectric biomaterial-based scaffolds for the repair and regeneration of dental tissues [11,12,13]. Indeed, such materials have shown tissue differentiation, antimicrobial, and anti-inflammatory properties. In this review, we first describe the basic principles and mechanisms of piezoelectricity. We then report a detailed bibliometric analysis of the published research on piezoelectric biomaterials used for tissue engineering. Then, we critically discuss promising piezoelectric biomaterials that could be exploited for engineering the dentin-pulp complex and the methods by which piezoelectric scaffolds may be fabricated.

**Table 1:** Biomarkers involved in the regenerative roles of piezoelectric biomaterials in various tissues.

Piezoelectric Biomarker	Role in Tissue Regeneration	Reference
Increased expression of transforming growth factor (TGF-β)	Voltage-gated Ca^2+^ ion channel opening	[15]
Increased expression of bone morphogenetic protein (BMP)	Bone remodeling	[15]
Increased expression of collagen type-3 (Col III)	Collagen synthesis and tissue granulation	[15]
Increased expression of collagen type 4 (Col IV)	Keratinocyte migration	[15]
Upregulation of CD68	Macrophage differentiation, anti-inflammatory	[16]
Upregulation of vascular endothelial growth factor (VEGF)	Anti-inflammatory	[15]
Upregulation of integrin α5	Angiogenesis	[15]
Upregulation of CD99	Angiogenesis	[15]
Extracellular signal-regulated protein kinase (ERK1/2)	Electrostatic migration	
Proliferating cell nuclear antigen (PCNA)	Cell proliferation and signaling	[15]
α-actin	Myofibroblastic differentiation	[15]
Rho-GTPase	Electrotaxis	[17]
Enhanced phosphorylation of PI3K	Electrotaxis	[17]
Enhanced phosphorylation of Akt	Electrotaxis	[18]
Downregulation of Scleraxis	Tenogenic differentiation	[19]
Runt-related transcription factor 2 (Runx 2)	Osteogenic differentiation	[20]
Myoblast determination protein (MyoD)	Terminal myogenic differentiation	[21]
Myocyte enhancer factor-2 (MEF2)	Myogenic differentiation	[21]

## 2. Basic Principles of the Piezoelectric Effect

The term ‘piezoelectric’ is derived from the Greek word ‘piezein’ meaning pressure. The preliminary hypothesis of the ‘piezoelectric effect’ was proposed by the renowned French physicists Jacques and Pierre Curie in 1880 [22]. However, it was not until 1946 that Cady [23] deciphered the underlying principle of the piezoelectric properties of barium titanate (BaTiO_3_, BT) and is recognized as the ‘Father of Piezoelectricity’ [24,25,26]. 

**Figure 2:**
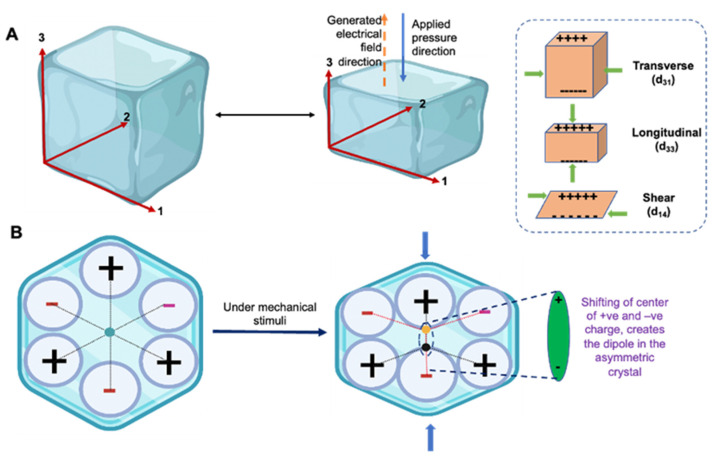
Fundamentals of the piezoelectric effect. (**A**) The direction of the piezoelectric coefficient, i.e., d_33_, implies that the generation of electric potential takes place in direction 3 in response to the applied stress from direction 3; (**B**) Mechanism of piezoelectric potential generation in a crystal.

The basic principle of the piezoelectric effect is the crystal habit deformation of a material under definite mechanical stress [26], which is attributed to their asymmetric crystal architecture and microcrystalline hierarchy. Typically, any material can experience mechanical stimuli from the external environment. This could be elongation or tension, twisting or shear, bending or torsional, and squeezing or compression forces, depending on the direction of the mechanical loads. Mechanical-stress-induced deformation causes a relative shifting of the positive and negative charge center in the material crystal architecture, generating the motion of an electric dipole or polarization. This aligned distorted electric dipole state leads to the generation of electrical potential and causes a charge flow. In contrast, in non-piezoelectric materials, the overall charge center of positive and negative ions in the unit cell coincides, and even with applied deformation, these electric dipoles cancel out, and no overall polarization appears (Figure 2). 

Piezoelectricity is a linear and reversible process. Transformation of a mechanical stimulus into electrical potential is termed the ‘direct piezoelectric effect’, whereas the reverse phenomenon is known as the ‘indirect piezoelectric effect’. Real-world applications of piezoelectric materials were limited to acoustic devices based on the ‘indirect piezoelectric effect’ in several healthcare devices, including ultrasound transducers and MRI contrast agents [27], until ‘direct piezoelectricity’ was demonstrated in quartz and Rochelle salt. It was further discovered that crystalline polymorphic materials such as barium titanate (BaTiO_3_), lithium niobate (LiNbO_3_), calcium titanate (CaTiO_3_), and strontium titanate (SrTiO_3_) exhibited a direct piezoelectric effect due to phase transition from one crystal structure to another, a phenomenon known as the ‘ageing effect’ [28]. For instance, barium titanate (BT) crystal shows phase transition from a non-piezoelectric cubic structure to a non-centrosymmetric tetragonal structure under mechanical stress. As piezoelectricity is an inherent physical property of the material (such as pyroelectricity and ferroelectricity), it loses its effect after heating past the ‘Curie temperature’ (T_c_). Different materials exhibit different Tc, which limits, in some cases, the real-world applications. For example, T_c_ for BT is 120 °C whereas for LiNbO_3_, it is 1140 °C, making the latter suitable for high-energy applications such as energy harvesting devices and tire pressure monitoring systems [29].

The piezoelectric coefficient (or piezoelectric modulus) is a material constant that relates the polarization per unit of the electric field experienced by the material per unit of the applied mechanical stress and is expressed in the unit of Coulomb/Newton (C/N). This coefficient is expressed as ‘d_ij_’, where ‘i’ indicates the direction of polarization in the material and ‘j’ indicates the direction of the applied stress (or induced strain). For example, in the constant d_33_, the generation of electric potential takes place in direction 3 in response to the applied stress from direction 3 (Figure 2), which means that the mechanical load is applied parallel to the polarization axis [27,28]. The piezoelectric charge coefficient is most frequently used to evaluate the goodness of a piezoelectric material. The piezoelectric coefficient has different magnitudes depending on the direction of the applied mechanical stress and polarization. In the case of d_31_, the charge is collected from the same surface as d_33_, but the force is applied at right angles to the polarization axis, rending a different constant value. For example, for BT, d_33_ is 90–788 pC/N whereas d_31_ is −33.4 to −78 pC/N [29]. 

The interactions of piezoelectric materials with biological processes in a mammalian cell is termed ‘bio-piezoelectricity’ [7,15]. Piezoelectric charges play a role in various physiological processes, including cell division, migration, differentiation, and regeneration. Notably, the effect of bio-piezoelectricity on cells is more prominent in tissues that undergo normal physiological movement or in stress-bearing organs [2,15] due to the proportional relationship between the piezoelectric potential and the applied stress. For example, tissues such as bone and cartilage are dynamically stimulated by functional loads, and piezoelectric scaffolds may stimulate the regenerative signaling pathways to enhance tissue regeneration at the impaired site [8]. However, the interaction between the dentin-pulp complex and piezoelectric biomaterials remains poorly investigated and is an important avenue for future research.

## 3. Bibliometric Analysis of Research on Piezoelectric Biomaterials for Tissue Engineering

To inform the status of piezoelectric-based biomaterials for tissue engineering, we performed a bibliometric analysis using data acquired from three databases (PubMed, Scopus, and SciFinder) based on relevant keywords (‘piezoelectric biomaterials’, ‘piezoelectric polymers’, ‘tissue engineering’, ‘bone, cartilage, neural, skin, and dental’). We then excluded reviews, systematic reviews, hypotheses, and literature-survey-based articles to ensure that only original research articles were included. To focus our sample space of the analysis on the direct applications of piezoelectric biomaterials on tissue engineering, we excluded research articles related to the topic of piezoelectric biosensors, implantable sensors, and drug delivery.

**Figure 3:**
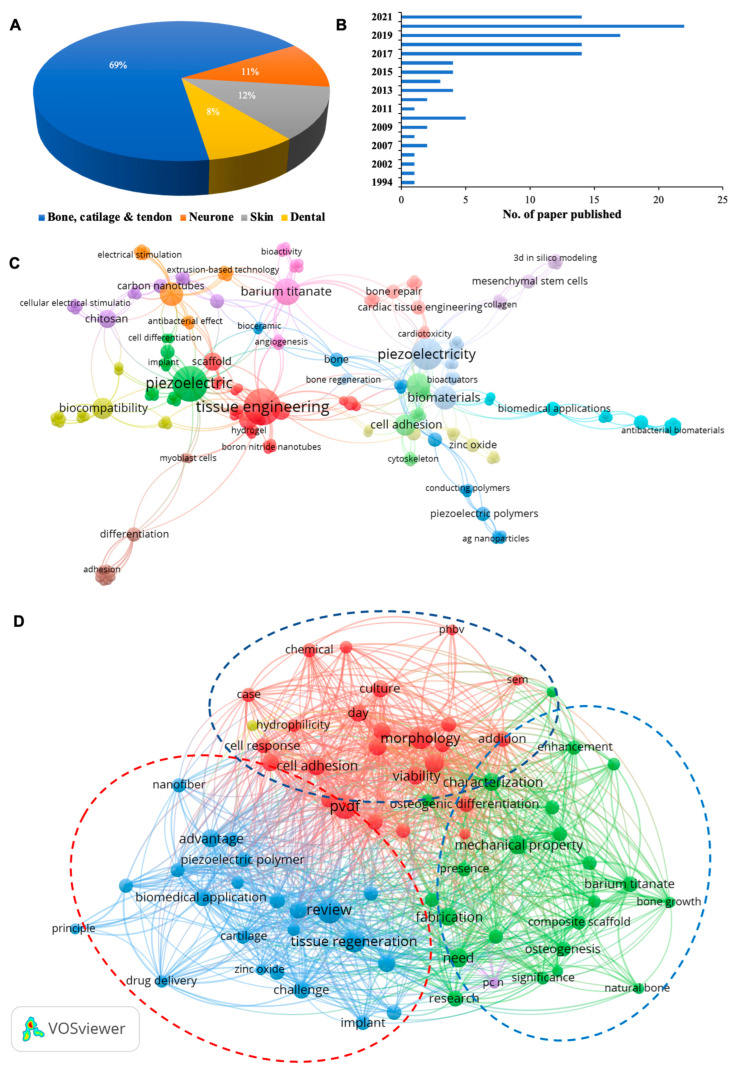

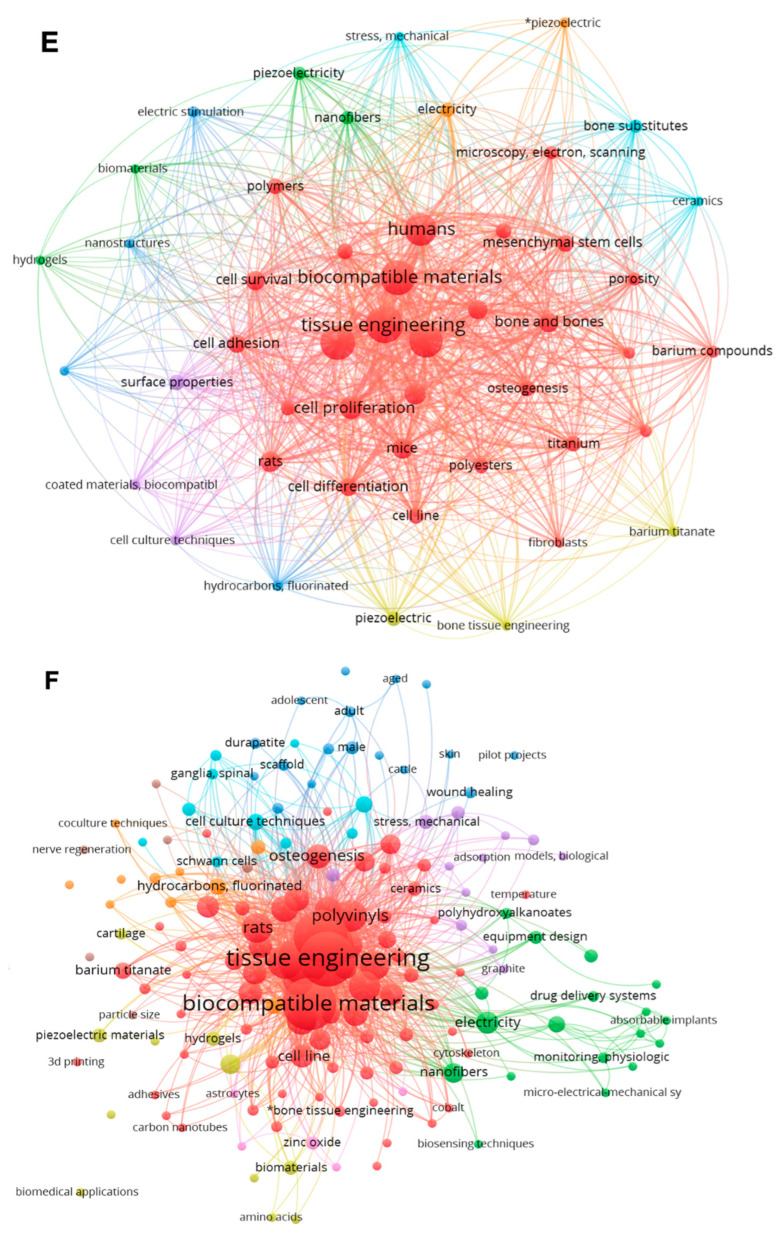
Bibliographic analysis of piezoelectric biomaterials for tissue engineering applications; (**A**) Pie chart of the percentage of articles published on tissue engineering applications of piezo-materials; (**B**) Year-wise number of studies published on piezoelectric biomaterials; (**C**) Bibliographic coupling analysis of piezoelectric biomaterials for different tissue engineering applications; (**D**–**F**) Bibliographic coupling analysis for piezoelectric scaffold for bone and cartilage tissue engineering and skin tissue, respectively (performed and schematic made using VOSviewer). The size and color of the nodes represent the number of references that are shared among the analyzed papers. The strength of a link indicates the number of cited references that the two publications have in common.

A total of 271 articles published from 1982 to 2021 formed the sample space. It was observed that from 1982 to 2021, 58% of the work on piezoelectric biomaterials was performed on bone, cartilage, and tendon tissue engineering while 13% and 10% of the work was performed on skin and neuronal tissue engineering, respectively. Notably, only 3% of the work was associated with dental tissue engineering (Figure 3). There was a continuous increase in the number of published research articles from 1982 to 2011 in piezoelectricity-based dental tissue engineering. We then used VOSviewer software to correlate the bibliometric data and visualize the interlinks between different piezo scaffolds with their biological properties. Most of the piezoelectric biomaterials were fabricated into three most preferred forms, i.e., nanofibers, hydrogels, and 3D-printed scaffolds composed of polymeric materials to support tissue repair and regeneration. In general, it was observed that studies on the application of piezoelectric biomaterials in engineering to a range of hard and soft tissues, including bone, cartilage, skin, and dental tissues such as pulp, periodontal ligament, and alveolar bone were closely linked to studying cellular responses such as adhesion, differentiation, and proliferation. Characterization of the mechanical strength and the ensuing mechanisms were explored for piezoelectric dental biomaterials.

VOSviewer is a bibliometric analysis tool used to correlate, construct, summarize, and visualize bibliometric data obtained from different publication databases [30,31]. The advantage of VOSviewer is that it can indicate the interactive relationship between a co-authorship network and bibliographic coupling analysis, which gives an overview to identify potential research hotspots for future research [32]. Here, we used VOSviewer software to correlate the bibliometric data and visualize the interlinks between different piezo scaffolds with their biological properties. 

After obtaining and creating the bibliographic library, we performed three bibliographic coupling analyses, as demonstrated in Figure 3C–F. Figure 3C,D demonstrate the bibliometric coupling analysis diagrams, where different piezoelectric biomaterials were explored for several tissue engineering applications and corelated with their reported cellular functionality. For example, most of the piezoelectric biomaterials were fabricated in the three most preferred forms i.e., nanofibers, hydrogels, and 3D-printed scaffolds composed of polymeric materials for the support of tissue repair and regeneration. In general, it was observed that the applications of piezoelectric biomaterials in tissue engineering to a range of hard and soft tissues were closely linked to the study of cellular responses such as adhesion, differentiation, and proliferation. 

Most studies explored the cell adhesion and proliferation behavior of piezoelectric-based nanofiber scaffolds, whereas other fabricated forms such as nanoparticles and hydrogels were explored for their superior biocompatibility and stem cell proliferation. Notably, there were limited studies on antimicrobial activity and related mechanisms. Interestingly, studies related to bone and cartilage tissue engineering mainly correlated with the mechanical strength, durability, osteoconduction, osteointegration, osteogenesis, and cell differentiation properties of piezo-materials (Figure 3E), whereas in the case of skin tissue engineering or wound healing applications, it was linked with the cell adhesion, proliferation, migration, and stem cell differentiation characteristics of biomaterial scaffolds due to the piezoelectricity (Figure 3F). The work carried out related to dental tissue engineering was also linked with the stem cell differentiation, mechanical strength, cell adhesion, proliferation, and antibacterial effect-based bioactive properties of piezo-materials. However, it is important to note that this bibliometric analysis was only based on scientific publications in the English language and herein, the sole intention herein is to offer a bird’s eye view of the overall research trends on piezoelectric-based biomaterials before delving deeper into the materials science in later sections. 

Piezoelectric responses in these studies were characterized using state-of-the-art approaches such as piezo force microscopy (PFM) [5] or PiezoMeter by measuring piezoelectric strain coefficients and piezoelectric charge constants (d), piezoelectric voltage coefficients (g), and electromechanical coupling coefficients (k). Among these, determination of the piezoelectric charge coefficients (d) and piezoelectric voltage coefficients (g) was the most common. It is also important to mention that piezoelectricity is a third rank tensor and thus all the coefficients are dependent on the directions of the applied pressure [27]. Taken together, this bibliometric analysis shows that there is emerging interest in developing piezo-material-based biomaterial scaffolds for multifunctional applications. However, future research should investigate the exact mechanisms underlying the tissue engineering responses of piezoelectric materials.

## 4. Piezoelectric Biomaterials for Engineering the Dentin-Pulp Complex

Over the years, many materials, including ceramics, polymers, doping elements, and synthetic amino acid/polypeptides, have been discovered to have piezoelectric properties. In this part of the review, we critically discuss piezoelectric materials that have potential to be developed for dental tissue engineering (Table 2).

### 4.1. Piezoelectric Ceramics

One of the earliest piezoelectric materials was lead-zirconium-titanate or PZT, which exhibits an easy dipole orientation and possesses a high piezoelectric coefficient of about 250–350 pC/N due to its ABO_3_ perovskites crystal structure (where A and B are cations) [40,41]. However, it is not suitable for biological applications due to the cytotoxic nature of Pb^2+^ ions [41,42] and hence is not discussed in this review. However, it is notable that lead-free piezoceramics such as Li-modified (Na,K)NbO_3_(LNKN), (Bi, Na)TiO_3_ (BNT), (Na,K)NbO_3_(NKN), and tungsten bronze (TB) have been developed to circumvent the challenge of toxicity, but their biological applications remain to be elucidated.

### 4.2. Barium Titanate (BT)

The piezoelectric property of barium-based ceramic materials was discovered during the poling of BT. Electrical poling or corona poling is the process of phase transition achieved by the application of a high electrical field (1–30 kV) at temperatures higher than the Curie temperature [19,41]. BT offers a superior advantage for biological use than other piezoceramics such as PZT due to its excellent biocompatibility even at concentrations >100 μg/mL [43]. The piezoelectric coefficient d_33_ of BT is <350 pC/N. The incorporation of BT nanoparticles in a PLGA polymer matrix improved its cell proliferation, osteoconductive, and osteointegration behavior in osteoblasts and osteocytes, favoring bone tissue engineering [43]. The incorporation of BT nanoparticles as a filler material in the traditional dental resin composite [44] showed antibacterial and remineralization effects. BT-incorporated denture polymers, such as polymethyl methacrylate, eradicated fungal biofilms and potently killed Candida albicans [16] due to the piezoelectric charges generated by the barium titanate nanoparticles during simulated masticatory forces, which resulted in the generation of reactive oxygen species (ROS) and consequent upregulation of the superoxide dismutase gene (SOD5) [16].

**Figure 4:**
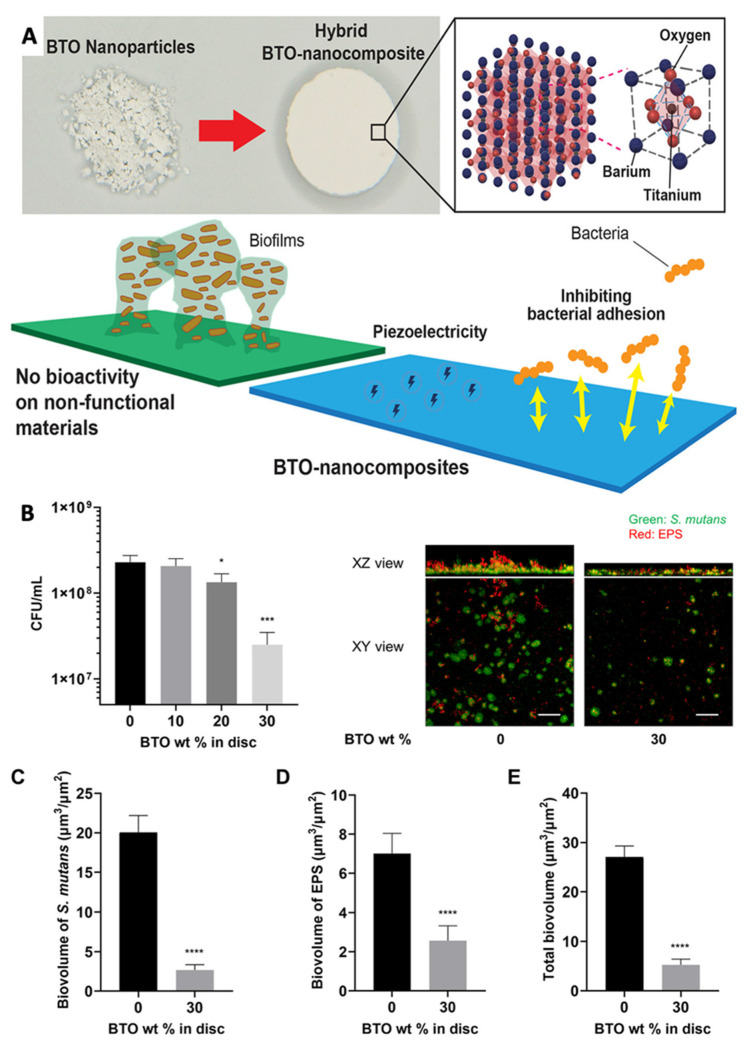
(**A**) Bimodal piezoelectric barium titanate nanocomposite exhibits antibiofilm activity; (**B**) Dose-dependent anti-biofilm activity and confocal images of *Streptococcus mutans* biofilms after 18 h on the BT/hydroxyapatite piezoceramic discs. (**C–E**) Quantified biovolume of *S. mutans*, EPS, and total biovolume (sum of *S. mutans* and EPS) in the biofilm, Statistics: *t*-test with *, *** and **** represents *p* < 0.01, *p* < 0.001 and *p* < 0.0001 respectively; Reprinted with permission from [17], Copyright 2021, [17], American Chemical Society, with minor modifications.

A piezoelectric BaTiO_3_-hydroxyapatite-based nanocomposite platform was reported by Dhall et al. [17] to overcome biofilm-associated infections and consequent failures in medical devices (Figure 4). They showed that the addition of piezoelectric BaTiO_3_ nanoparticle in a hydroxyapatite scaffold resulted in dose-dependent activity against *Streptococcus mutans* biofilms. They observed a 10-fold reduction in colony-forming units (CFU) compared to the pristine scaffold. Furthermore, it was demonstrated that an increase in the concentration of the piezoelectric BaTiO_3_ nanoparticles from 10 to 30 wt% in the scaffold increased the negatively charged surface energy, creating an unfavorable condition for bacterial adhesion [17]. Nonetheless, Fan et al. reported the angiogenetic capability of BT under the influence of ultrasound waves in a titanium implant coated with BT nanoparticles for large segmental bone defects [19]. Therefore, having multifunctional properties (antibacterial, antibiofilm, anti-inflammatory, mineralization, and angiogenesis), long-width piezoelectric BT-based piezo-platforms have great potential for further research on regeneration of the dentin-pulp complex.

### 4.3. Zinc Oxide (ZnO)

Despite the well-explored biological applications of ZnO due to its antibacterial property, biocompatibility, and its favorable role in cell adhesion and differentiation, the investigation and application of its piezoelectric properties remain limited. ZnO crystals demonstrate a piezoelectric d_33_ coefficient of 3.62 ± 0.5 pC/N [20] because of their hexagonal asymmetric wurtzite crystal structure and polarized crystal surface [19,21]. A recent study by Bhang et al. [5] demonstrated the effect of piezoelectric ZnO nanorods in wound healing and skin tissue regeneration in both in vitro and in vivo mice models. They developed a multi-layered dermal patch by reinforcing a polydimethylsiloxane (PDMS) matrix with ZnO nanorods through pin coating. Mechanical rubbing with a soft velvet cloth was used to induce the directional alignment of the dipole. By varying the concentration of ZnO nanorods to 54.8% and 95.2%, they obtained a range of piezo potentials from 300 to 900 mV [5]. The fabricated patch significantly increased piezoelectric biomarkers, including PCNA, TGF-β, COL-III, COL-IV, and α-actin. It was also shown to upregulate several marker genes such as CD68, VEGF, and CD99 in an athymic mice model. Taken together, these findings signify that the piezoelectric charges from ZnO are involved in various molecular pathways that regulate cell migration, tissue granulation, cell proliferation, and differentiation, which are essential for skin regeneration.

A study on the size-dependent cytotoxic behavior of ZnO [18,21] reported that macro- and micro-range (>1 µm) ZnO particles do not have any toxicity, but nanoparticulate ZnO (i.e., 20–200 nm) exhibits cytotoxicity above a 0.2 µg/mL concentration in squamous cell carcinoma (HNSCC) and HepG2 cells in vitro due to significant reactive oxygen species (ROS) generation [45,46]. Nevertheless, this drawback could be overcome by surface and chemical modifications. For instance, Ramasamy et al. [46] found that a thick coating of SiO_2_ on the surface of 20 and 50 nm sphere-like ZnO NPs improved its cytocompatibility on human skin dermal fibroblast neonatal (HDFn) cells compared to bare ZnO NPs [46]. Furthermore, the modified hydrophilic surface of the SiO_2_ coating stabilized the ZnO NPs due to loosened aggregation. The coated ZnO NPs had a lesser degree of LDH leakage, ROS production, and LPO release compared to pristine ZnO NPs [46]. However, the impact of these surface modifications on the piezo property of nanoparticles remains unknown.

### 4.4. Piezoelectric Polymers and Composites

Piezoelectric polymers have several advantages over ceramic- and metal-based piezoelectric materials such as facile fabrication, broad tunability of physiochemical properties by surface functionalization, chemical modification, and the ability to fabricate different types of scaffolds such as nanoparticles, nanofibers, nanorods, hydrogels, composites, and cryogels [18,29]. Moreover, their low cytotoxicity and physiological roles such as cell adhesion and proliferation [5], stem cell differentiation [47,48], and ultrasound-mediated mechano-transduction ability [49] endow them with unique advantages in biomedical applications.

#### 4.4.1. Synthetic Polymers

- Polyvinylidene fluoride (PVDF) and its derivatives

PVDF has been used as the ‘gold standard’ piezoelectric polymer for various tissue engineering applications. Along with its piezoelectric property, PVDF has unique advantages such as antioxidant behavior, high strength, and high thermal, chemical, and hydrolytic stability [3,48]. PVDF exhibits piezoelectric properties due to its polarized molecular structure. The general molecular formula of PVDF is (-CH_2_-CF_2_-) n. Typically, PVDF exists in five polymorphic forms, namely α, β, γ, δ, and ε phases, depending on the nature of the tacticity of the hydrogen and fluorine atoms [50]. Among these, only the β phase shows inherent piezoelectricity due to its all-trans molecular configuration and in this atomic configuration, all the -CH_2_- dipoles are perpendicular to the -CF_2_- repeat units, which results in an inherent electric dipole moment [50].

Several techniques such as corona poling, uniaxial and biaxial drawing, high voltage electrospinning, and annealing facilitate the phase transformation and increase the β phase concentration in PVDF [16,51]. PVDF shows an average piezoelectric d_33_ coefficient of 20 pC/N [48] due to the presence of highly electronegative F atoms and its trans-gauche-trans-gauche’ (TGTG’) atomic configuration in a centrosymmetric unit cell [51]. The negatively charged surface of PVDF scaffolds is induced through the corona poling process to promote better cell adhesion and proliferation in C2C12 mouse myoblast cells compared to non-poled PVDF specimens for skeletal muscle regeneration [52]. More recently, it was demonstrated that poled β-PVDF samples have better protein adsorption and osteogenic differentiation on human adipose stem cells (hASCs) compared to unpoled scaffolds [53]. It was also reported that PVDF fibrous membrane as a scaffold for growing and recapitulating the multi-layered chondrocytes resulted in significantly greater gene expression for fibronectin and integrin α-10 in chondrocytes that adhered to the PVDF surface [53]. Furthermore, this study demonstrated that chondrocyte cell sheets had a similar phenotype to the regular ones and had increased gene expression of SOX9 and Col XXVII [53]. In a different study, the mineralization potential of PVDF scaffolds was revealed, with the amount of formed mineral proportional to the magnitude of external mechanical stimulation [54].

**Figure 5:**
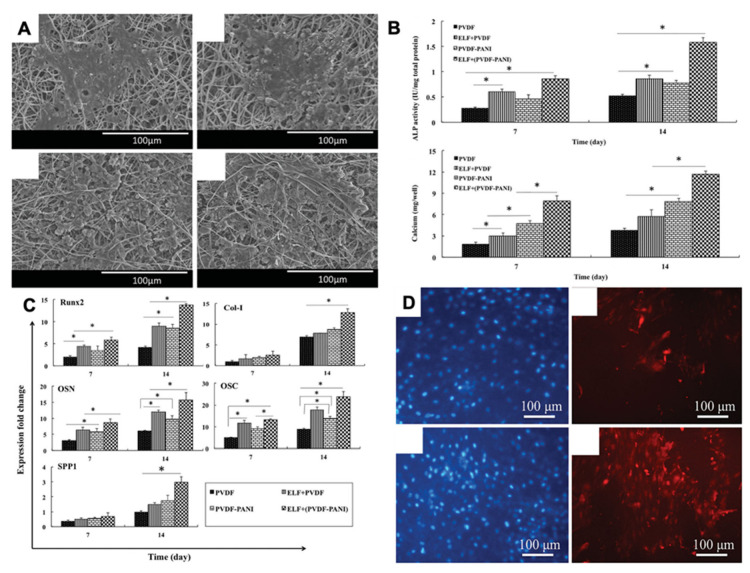
(**A**) SEM images of DPSC-seeded electrospun PVDF scaffold after PEMF exposure; (**B**) Alkaline phosphatase and calcium content assays of the differentiated DPSCs under osteogenic medium; (**C**) Different relative gene expression of DPSC-seeded electrospun PVDF scaffold at 7 and 14 days in the absence and presence of PEMF, the significant differences (*p* < 0.05) between groups are indicated with * sign; (**D**) Immunocytochemistry (ICC) staining for osteocalcin protein in the differentiated DPSCs. Reprinted with permission from [56]. Copyright 2019 [56], Taylor & Francis Online, with minor modifications.

Two-dimensional and three-dimensional PVDF nanofiber scaffolds show differential effects on human-induced pluripotent stem cells (iPSCs) [34]. iPSCs seeded on 3D fibrous scaffolds showed significantly greater osteogenic-related genes and protein expression compared to the 2D scaffold, resulting in superior osteoinductive effects and better bone differentiation. Another remarkable study by the same group reported the potential of PVDF-polyaniline (PANI) piezoelectric electrospun scaffold for the differentiation of stem cells derived from dental pulp (DPSCs) [55]. To the best of our knowledge, this is the only study to date that has indicated the effect of piezoelectricity on the osteogenic differentiation of dental stem cells. This study applied a low-frequency pulsed electromagnetic field (PEMF) as a source of mechanical stimulation for the piezo-scaffolds and observed that DPSCs seeded on the scaffold exhibited better cell adhesion, increased alkaline phosphatase activity, a higher calcium content, and significantly higher osteogenic gene expression compared to unexposed samples (Figure 5) [56].

Chemical derivatization of pristine PVDF polymer such as P(VDF-TrFE), a copolymer of vinylidene fluoride (VDF) and trifluoroethylene (TrFE) [57,58], results in an enhanced electromechanical conversion compared to PVDF [59]. For instance, Hunage et al. reported that biaxially oriented poly (vinylidene fluoride) exhibits a higher piezoelectric coefficient (d_33_ = −62 pC/N) than pristine PVDF [60]. Electrospun nanofibrous mats of P(VDF-TrFE) were shown to regenerate neuronal tissue from PC-12 cells upon ultrasound stimulation [4]. Notably, the neurite outgrowth was uniform in all directions on the piezo scaffolds compared to the neural growth factor added media. It has also been demonstrated that human mesenchymal stem cells (MSCs) cultured on a thermally poled piezoelectric PVDF-TrFE nano-fibrous scaffold exhibited tissue-specific chondrogenesis and osteogenesis as confirmed by the GAG content, collagen type II to I ratio, ALP-mediated mineralization, and osteogenic gene expression [61].

Dental pulp stem cells (DPSCs) grown on PVDF-polyaniline piezoelectric nanocomposite scaffolds were shown to have improved osteoinductive capability. This study demonstrated a higher level of DPSC adhesion, alkaline phosphatase activity, calcium content, and osteogenic gene expression under a low frequency pulsed electromagnetic field (PEMF) [56]. Therefore, blending a conductive polymer such as polyaniline with a piezoelectric polymer could be a useful approach for improving cell attachment and stem cell differentiation while it remains unknown whether the cells can be differentiated to preferred phenotypes by modifying the piezoelectric behavior.

**Figure 6:**
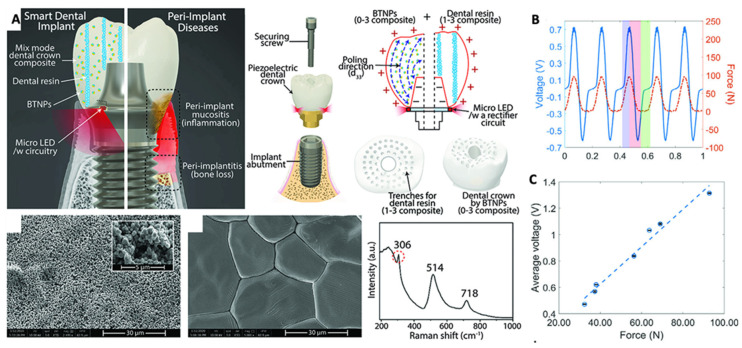
(**A**) Schematic illustration and SEM images of Piezoelectric Smart Dental implants (SDIs) capable of generating light under physiological chewing and brushing motion for photo-biomodulation therapy; (**B**,**C**) Electrical voltage generated from the piezoelectric-SDI under chewing motion (the applied force was ≈90 N at a frequency of 5 Hz) and average voltage outputs of SDI under soft food chewing motions that ranges from 30 to 100 N (f = 5 Hz). Reprinted with permission from [62], Copyright 2022, [62], Wiley Online library, with minor modifications.

Multifunctional piezoelectric-based smart dental implants (SDIs) composed of PVDF and BaTiO_3_ have been shown to exhibit anti-inflammatory activity in addition to its concomitant use with photo-biomodulation (PBM) therapy by energy generated from normal human oral motions such as chewing and brushing (Figure 6). SDI was shown to exhibit higher cell viability and anti-inflammatory activity upon harvesting the electrical charge that accumulated on the piezoelectric scaffold [62]. This work demonstrated that the piezo scaffold was able to generate light in situ for photo-biomodulation (PBM) therapy, which regenerates and restores the damaged peri-implant soft tissue. Traditional NIR-based photodynamic therapy (PDT) for peri-implant soft tissue regeneration requires 0.8 V electrical stimulation, which can be generated from the piezoelectric scaffold from 60 N mechanical pressure of chewing motion or 90 N of brushing motion [62]. Thus, this groundbreaking study set the stage for integrating multifunctional and smart therapeutics using piezoelectric materials in oral healthcare. 

Moreover, all the above findings indicate that PVDF serves as a center of attention of piezoelectric materials due to its ease of processability, stable piezoelectric response, and good biological characteristics. However, limited attention has focused on tuning the chemical properties of PVDF instead of blending with other materials; for instance, surface functionalization or conjugation of biomolecules could be a better approach to explore the multifunctionality of PVDF-based piezo scaffolds. We believe that the next-generation bio-piezoelectric platforms can be developed by strategically combing the physical piezoelectricity with the chemical properties of piezoelectric biomaterials.

- Poly-L-Lactic Acid (PLLA)

**Figure 7:**
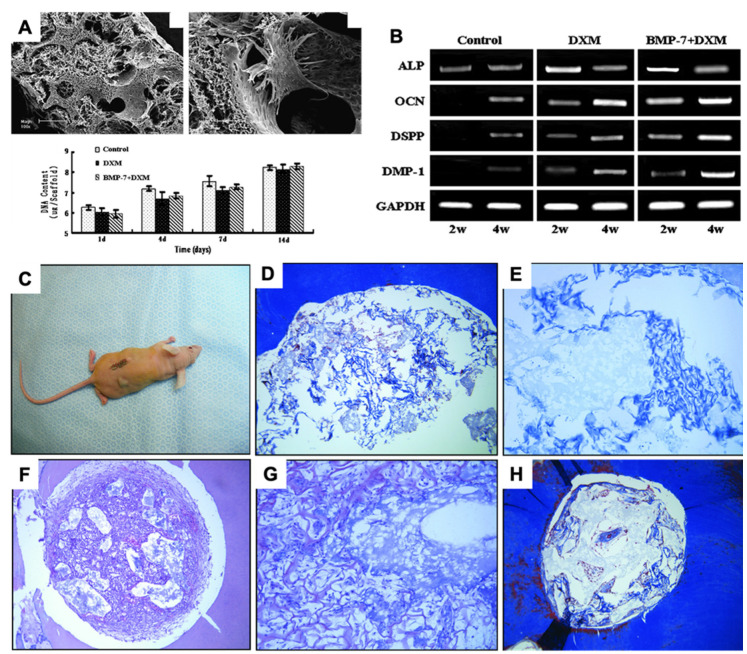
(**A**) SEM images of PLLA-nanofibrous scaffolds loaded with DPSCs cultured in vitro for 3 days and proliferation characteristics of DPSCs; (**B**) Gene expression of DPSCs grown on PLLA scaffolds for 2 and 4 weeks. Reprinted with permission. Copyright 2010 [41] Elsevier, with minor modifications. (**C**–**H**) Histological images of collagen scaffold, DPSCs, and DMP1 scaffold constructs implanted in vivo for 8 weeks. Reprinted with permission from [35]. Copyright 2018 [35], Journal of Endodontics, with minor modifications.

PLLA is one of the best-known biodegradable polymeric biomaterials used for dental tissue engineering [63,64]. There are four polymorphic forms of PLLA—α, β, δ, and γ phase, all of which exhibit piezoelectricity [65]. The helical topography and quasi-crystalline nature are responsible for the piezoelectric property of PLLA, resulting in a piezoelectric shear coefficient (d_14_) of −10 pC/N [65,66]. Electrospun nanofibers of PLLA have been shown to induce DPSCs’ differentiation into mature odontoblasts (Figure 7A,B) and recapitulate the dentin-pulp histoarchitecture in vitro [48]. However, whether the piezoelectric properties of PLLA contribute to such effects remains unknown.

An advantage of the PLLA-based piezoelectric scaffolds is that they do not require additional electrical or mechanical poling to enable piezoelectricity. Comparing the proliferation and maturation of DPSCs on various scaffolds, Chandrahasa et al. showed that this nonpolling requirement capability is influenced by the chemical composition of the scaffolds and that PLLA scaffolds showed greater mineralization ability than bovine collagen and calcium phosphate scaffolds [67]. A recent study by Das et al. [68] showed the osteogenic differentiation ability of piezoelectric PLLA nano-fibrous scaffolds from adipose stem cells (ADSCs) and bone marrow stem cells (BMSCs) under ultrasound stimulation. They reported that a piezoelectric PLLA scaffold with ultrasound stimulation exhibited greater collagen 3.6 gene expression in a bone defect mice model than without ultrasound. It is noteworthy to state that the upregulation of the collagen 3.6 gene, which is normally present in mature osteoblasts, directly implies greater osteoblastic activity. Moreover, PLLA-based scaffolds show controlled biodegradation and excellent biocompatibility. However, it is noteworthy that the biodegradation of PLLA forms lactic acid, which may have a negative impact on the mineralization of dental hard tissue [69].

- Poly-3-Hydroxybutyrate-3-Hydroxyvalerate (PHBV)

PHBV has received considerable attention due to its accepted biocompatibility, biodegradability, and appropriate mechanical properties and thermoplasticity for biomedical applications [70]. Interestingly, PHBV also possesses a piezoelectric d_33_ coefficient of 1.3 pC/N, which is almost equivalent to type 1 collagen in human bone [71]. Due to these characteristics, PHBV-based piezoelectric scaffolds have been investigated for bone tissue engineering. Kose et al. reported the cartilage tissue regeneration ability of collagen-PHBV scaffolds [71]. A study by Jacob et al. [72] showed that the nano-fibrous scaffolds of BT-nanoparticle-reinforced PHBV composites exhibit higher chondrocytes and Col-II gene expression activity. They also pointed out that the reinforcement of BT-NPs as a filler increases the strength of the pristine PHBV scaffold up to 20%. This study indicated that it is possible to regenerate or repair any tissue without using any type of chemical or biological factors such as growth or transcription factors by solely utilizing piezoelectric scaffolds. 

#### 4.4.2. Natural Biopolymers

- Collagen

Being the most abundant natural biopolymer, collagen is considered one of the best biomaterials for tissue engineering applications, including dental tissues [73,74]. It is known that collagen type I in the dental matrix serves as the base material for the calcification of dental tissue [75,76]. The piezoelectric shear coefficient (d_15_) of collagen type I fibril was found to be 0.51 pm/V, as quantified by PFM [77].

There is evidence that demonstrates that collagen scaffold induces the regeneration of odontoblasts and encourages the formation of odontoblasts and adhesion with the pulp [78]. Over the past few years, numerous studies have reported a multitude of applications of collagen scaffolds, collagen gels, and sponge in dentin-pulp complex regeneration, stem cell differentiation, and cellular proliferation [35]. One such study [35] demonstrated that after 6 weeks of seeding DPSCs on a collagen substrate, a physiological-mimicking matrix architecture was formed (Figure 7C–H). In addition, a DPSC-seeded 3D collagen scaffold can proliferate and differentiate into odontoblasts [79]. However, they did not decipher the role of piezoelectricity in this differentiation. Piezoelectric collagen-hydroxyapatite composites not only have sufficient mechanical strength, biocompatibility, and low antigenicity but also exhibit better cell adhesion, proliferation, and bone healing properties [80]. As a functional piezoelectric biomaterial, collagen-based piezo-scaffolds can provide the merits of good biocompatibility and degradation, but it also true that most collagen-based scaffolds have the burden of a stable piezoelectric response and the direction dependability of piezo-response of the collagen fibers also has a great impact on the clinical translation. Future research should be focused on the tunability of collagen-based piezo-scaffolds as these aspects can make it a gold standard piezoelectric material from biological origins.

- Chitosan

Chitosan is an abundant biopolymer, generally obtained from the deacetylation of chitin, which is found in the exoskeleton of insects and mollusks [81]. It is a remarkably well-explored biomaterial in various applications, including drug delivery, tissue engineering, wound healing, and biosensors [36,82]. Nevertheless, to facilitate an improvement in the mechanical and biological properties, chitosan is often blended with other materials. For instance, chitosan-carboxymethylcellulose (CMC) hybrid scaffolds demonstrated enhanced proliferation and significantly higher gene expression of osteonectin and dental sialophosphoprotein compared to a native chitosan scaffold [83]. Yang et al. reported distinct dental stem cell adhesion, proliferation, and differentiation properties of a BMP-7-loaded chitosan-collagen scaffold [74,84]. In addition, Liao et al. developed a bioactive chitosan scaffold with β-tricalcium phosphate (TCP), which promoted the vascularization of human periodontal ligament cells (HPLCs) in vivo [85].

Chitosan nanoparticles have also been added to endodontonic sealers for antibacterial purposes. For example, the incorporation of 2% wt/vol. chitosan nanoparticles into three commercial endodontic sealers (AH Plus-Dentsply DeTrey, Konstanz, Germany), Apexit Plus-Ivoclar Vivadent, Schaan, Liechtenstein, and MTA Fillapex-Angelus, Londrina, Brazil) resulted in superior antifungal activity than the sealers used alone [86]. Detailed investigations of chitosan nanoparticles by Kishen’s group [87] demonstrated their antibacterial and antibiofilm efficacy using dentin infection models. It was shown that polycationic chitosan nanoparticles interact with the negatively charged bacterial cell surface to cause bacterial killing and eradication of biofilms [87]. Excitingly, chitosan was recently shown to possess piezoelectric characteristics due to its ortho-rhombic crystal structure with the P2_1_2_1_2_1_ space group and non-centrosymmetric attributed to the glucosamine monomer [88]. Chitosan exhibits a range of piezoelectric d_33_ coefficients from 0.2 to 1.5 pC/N, depending on its source and degree of deacetylation [85,89]. Despite such promising findings in the above works, a cause–effect relationship between tissue regeneration and piezoelectric behaviors has not been reported for chitosan.

- Cellulose

Cellulose is the most abundant natural polymer, which exhibits excellent sustainable properties such as biodegradability, eco-friendly, low-cost production, excellent biocompatibility, and outstanding mechanical properties [90]. For these reasons, cellulose was explored as a center of attention to decipher its piezoelectric capability. Generally, cellulose is composed of a linear chain of glucose molecules with three side hydroxyl groups (-OH), with the glucose moieties connected by a β-1,4-glycosidic linkage [91]. The strong H-bonds between the side -OH groups give a unique preposition to the cellulose moieties to form a highly ordered crystalline structure. Usually, cellulose crystals exist in four different forms, i.e., cellulose I, II, III, and IV [92,93]. Among them, the most common naturally originating cellulose I is present in two different polymorphs forms: triclinic type I_α_ and monocline type I_β_, depending on the source of the extraction [94,95]. Interestingly, another type of crystal, cellulose II, which is monoclinic in nature, is converted into cellulose I by dissolution and alkali treatment [90]. The piezoelectricity of cellulose results from the net dipole moment of polar -OH groups present in the triclinic type I_α_ polymorph, which is arranged in a non-centrosymmetric order [95]. The piezoelectric property of cellulose was first explored by Fukada [96]. It was reported that the longitudinal piezoelectric d_33_ coefficient of natural cellulose is about 0.4 pC/N [37].

Recently, cellulose, which is extracted from bacterial species, has been reported to possess excellent mechanical properties, high water holding capability, and outstanding suspension stability features [97]. For instance, An et al. fabricated a bacterial cellulose membrane for the guided bone regeneration (GBR) using electron beam irradiation techniques [98]. They demonstrated that electron irradiation of a bacterial cellulose membrane resulted in enhanced in vitro cell viability, adhesion, and proliferation in NIH3T3 cells and in vivo bone regeneration on rat calvarial defect models compared to non-irradiated samples [98]. Another study [99] reported that reinforcement of micrometric particles of cellulose into silicate dental cement resulted in a shorter setting time, enhanced compressive strength, and enhanced cell adhesion and proliferation [99]. However, cellulose remains to be exploited as a piezoelectric material for the regeneration of dental tissues.

- Silk Fibroin

Being a natural biopolymer with good biocompatibility, excellent mechanical strength, and controlled biodegradation properties, silk has been explored as a multifunctional biomaterial for different biomedical applications, including drug delivery, tissue engineering, and regenerative medicine. For example, Woloszyk et al. [100] reported the neovascularization ability of DPSCs and gingival fibroblasts on a silk fibroin-based scaffold. They demonstrated that both cells have equal affinity to the formation of attracting blood vessels towards the damaged tissue microenvironment [100]. Jiang and coworkers [38] also investigated the proliferation and differentiation properties of DPSCs over a 3D-printed collagen/silk fibroin scaffold. They reported that after 1–5 days of incubation, it was observed that DPSCs seeded on a scaffold exhibited better cell adhesion and enhanced ALP activity, which induced DPSCs differentiation [38]. Kweon et al. [101] studied the effect of the addition of silk fibroin and hydroxyapatite coating on dental implants. They observed that after 6 weeks of implantation in a rabbit tibia model, the combined silk fibroin and hydroxyapatite coating groups had more new bone formation and bone-to-implant contact compared to uncoated and only hydroxyapatite-coated implants [101]. Nevertheless, a biomimetics approach was investigated by Huang et al. [102] for the creation of biominerals using a combination of spider silk and dentin matrix protein 1 (DMP-1). To achieve this, a novel spider-like domain and a domain of DMP-1 were cloned and expressed, and the two domains were then used for self-assembly and nucleation of hydroxyapatite [102].

Natural silk fibroin is a special type of block copolymer composed of two different heavy (~370 kDa) and light (~26 kDa) chains linked by disulfide bonds [103]. The heavy chain consists of alternating hydrophobic, repetitive oligopeptides that are separated by smaller charged and amorphous sequences. The hydrophobic domain is rich in alanine and glycine amino acids while the hydrophilic spacers give the heavy chain a polyelectrolyte nature [104]. Naturally, silk fibers are available in two different polymorphic forms, i.e., silk I and silk II [105]. Among them, silk II is present as a pleated, antiparallel β-sheet secondary structure with a monoclinic unit cell. The piezoelectric potential of the silk fibroin fibers mainly originates from its β-sheet content of silk II polymorphs. The uniaxially oriented polycrystalline silk fibers exhibit shear piezoelectricity, which indicates that the silk fibers generate electricity upon the exposure of certain shear stress perpendicular to its orientations [104,105]. A recent study by Yucel and colleagues [39] reported evidence of the structural origin of the piezoelectricity of silk fibroin. They evidenced that silk fibers exhibit a shear piezoelectric coefficient d_14_ = −1.5 pC/N after processing using the zone drawing method [39]. They also reported a correlation of the β-sheet content with an increasing draw ratio and the simultaneously increasing degree of the orientation of β-sheet crystals [39]. Thus, with the synergy of regeneration, mineralization capability, and piezoelectric potential generation, silk fibroin could serve as a potential piezo-biomaterial for dentin-pulp complex regeneration.

### 4.5. Amino Acids, Polypeptides, and Proteins

Intrinsic polar groups such as amino (-NH_2_) and carboxylic acid (-COOH) in the molecular structure of natural amino acids endow them with a unique advantage to exhibit piezoelectricity [39]. Most amino acids have been shown to possess piezoelectric potential due to their distinct crystal habits such as a right-handed (D) or left-handed (L) form [106]. Except for glycine, all amino acids have a chiral center, which results in the formation of non-centrosymmetry in the crystal lattice of the amino acids. This non-centrosymmetry of the groups results in the generation of piezoelectric tensors. However, most of the L or D-form amino acids exhibit shear piezoelectric tensors such as d_14_, d_25_, and d_36_ rather than longitudinal piezoelectric tensors such as d_11_, d_22_, or d_33_ [106]. It is important to note that the possession of a longitudinal piezoelectric coefficient is essential for real-world biological applications since real-life mechanical forces are perceived either in the compressive or tensile direction. Thus, attempts have been made to fabricate unidirectional and longitudinal piezoelectric amino acid crystals.

The extracellular matrix of mammalian tissues also comprises electroactive nano-crystalline polypeptide or protein molecules such as keratin, collagen, elastin, and glycosaminoglycans [107,108]. Piezoelectric phenomena in keratin were first reported by Martin when he observed the generation of a static electric potential from a bundle of wool (which is primarily composed of keratin) compressed within two brass plates. Keratin exhibits piezoelectric characteristics due to its highly ordered α-helical structure and the dipole originates from the hydrogen bond presence between the amine (-NH_2_) and carbonyl (-C=O) groups [7].

Recently, Guerin et al. studied the presence of a longitudinal piezo-response in β and γ-glycine due to their orthorhombic crystal orientation and validated the piezoelectric coefficient (d_16_) in β-glycine of about 2 × 10^2^ pm·V^−1^ [109]. They also reported a drop-casting solution-based facile fabrication method for the fabrication of amino acid crystal films with longitudinal piezoelectricity [109]. Density functional theory (DFT) based computational calculation was used to quantify the theoretical value of piezoelectric coefficients for several orthorhombic L-amino acid crystals (Table 3) such as threonine, asparagine, glutamine, histidine, proline, methionine, and isoleucine [110,111,112].

## 5. Fabrication Methods/Delivery Strategies for Piezoelectric Materials

### 5.1. Electrospun Fibers

**Figure 8:**
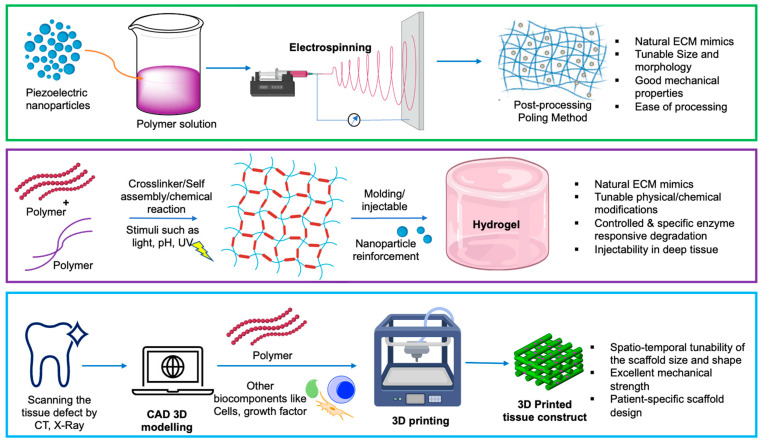
Different methods for the fabrication of piezoelectric biomaterial scaffolds.

Electrospinning is one of the most explored fabrication techniques for the development of nanofibers of piezoelectric materials (Figure 8). Nanofibers are popular scaffolds owing to its high surface area, tunable fiber morphology, ability to tailor the scaffold shape and fiber orientation, ease of surface functionalization, and porous structure, which mimics the natural extracellular matrix [116]. The underlying principle of electrospinning relies on the electrostatic interaction between a concentrated polymer solution and an oppositely charged collector system within a high voltage (1–30 kV) electrical field. Due to the high electric field, the surface tension of the polymer solution decreases and results in surface charge generation followed by Taylor cone formation, which leads to the stretching of the concentrated polymer solution and the formation of nanofiber deposited onto the collector system [117]. The ability to tailor the fiber diameter and morphology depends on several key factors such as the polymer concentration/viscosity, applied voltage, tip to collector distance, flow rate, needle diameter, temperature, and humidity. Different types of collector systems are also used to obtain different alignments and specific geometry of nanofibers; for instance, a commonly used rotating collector is used to align nanofibers in a parallel manner depending on its rotating speed [115,116].

Over the years, a plethora of works have reported the use of electrospinning scaffolds in dental applications, including pulp-dentin complex regeneration [118], repair of defects in periodontal tissues such as alveolar bone and periodontal ligament (PDL) [119], and guided tissue regeneration (GTR) membranes [120]. Nevertheless, it is also well evidenced that the reinforcement of nanoparticles with electrospinning scaffolds leads to multifunctionality and increased mechanical properties and tunable biodegradability. One such study reported by Bae et al. demonstrated that an electrospun scaffold of collagen with nano-bioactive glass (nBG) exhibited enhanced cell adhesion and proliferation, better mineralization, and increased levels of odontoblastic gene expression, including DSPP, DMP-1, ALP, OPN, and OCN, which leads to odontogenic differentiation of hDPSCs for dental-pulp tissue regeneration [116]. In another work, Guo et al. fabricated a polyurethane/polyvinylidene fluoride (PU/PVDF) electrospun scaffold for wound healing [121]. They reported that during the electrospinning process, the high electrical field induces a piezoelectric β-crystalline phase of PVDF [121]. Nevertheless, sometimes additional post-processing modification such as corona poling is also carried out to improve the overall piezoelectric performance and increase the d_33_ coefficient. For instance, Das et al. [68] improved the piezoelectric coefficient through the thermal poling process by annealing piezoelectric nanofibers at 105 °C for 10 h. Moreover, the process of electrospinning followed by thermal/electrical poling is a convenient technique to fabricate nanofibers of various piezoelectric polymer materials with diverse applications.

### 5.2. Hydrogels

A hydrogel is a three-dimensional network of a hydrophilic polymer system with more than a 10% water content in its structure, which in turn shows good biocompatibility due to the high moisture content [122] (Figure 8). Owing to its 3D matrix configuration, it not only mimics the natural ECM microenvironment but also supports cell attachment, proliferation, differentiation, regulating cell behavior, and intracellular signaling, simulating the recovery of the microenvironment of cell life properties [123,124]. A recent review by Ye et al. [124] reported the versatility of hydrogel scaffolds for several dental purposes such as dental pulp regeneration, periodontal tissue regeneration, and drug delivery. Another recent study by Siddiqui et al. [122] demonstrated the potential of self-assembled peptide hydrogel scaffolds for dental pulp tissue regeneration after pulpectomy, which is a major clinical challenge in endodontics.

Li et al. [123] fabricated a piezoelectric hydrogel of polyacrylonitrile-acrylamide-styrene sulfate-poly (vinylidene fluoride) (PAAN-PVDF) and reported that the fabricated PAAN-PVDF (15%) hydrogel was able to produce an output voltage close to 50 mV when the force was greater than 35 N [123]. However, it is important to note that in the case of the hydrogel matrix system, the output electrical signal resulted from two different aspects: one is the dipole moment of the piezo-material and the shape variable generated from the deformed hydrogel under certain forces. PAAN-PVDF also exhibits large stretchability (∼380%) and skin-like ductility and promotes angiogenesis in HUVEC cells under piezoelectric stimulation [123]. Another piezoelectric hydrogel was created by Zhou et al. [123] by combining poly (2-hydroxyethyl methacrylate) (PHEMA) doped with conductive nanoparticles, i.e., graphene oxide (GO) and single-walled carbon nanotubes (SWCNTs). They reported that the piezoelectricity of the resulting hydrogel is directly proportional to the amount of GO reinforcement but has a negative impact in the case of SWCNTs due to the internal slipping within a bunch of the nanotubes [123].

### 5.3. Additive Manufacturing: ‘3D Printing’

The exploration of additive manufacturing or 3D printing has been revolutionizing the worlds of tissue engineering and regenerative medicine (Figure 8). Three-dimensional printing gives us unique advantages over other conventional fabrication techniques thanks to its precise patient-specific complex anatomical replica fabrication with minimal efforts [125,126]. In dental applications, the usability of additive manufacturing technology has been explored, from orthodontics, periodontics, and restorative dentistry to endodontics and implant dentistry. For dental applications, different types of 3D printing platforms have been explored for different purposes such as stereolithography (SLA) or fused deposition modeling (FDM), which is mainly used for the fabrication of solid scaffolds, photopolymer jetting, and digital light processing using light cure resins, which have mainly been investigated for tissue engineering and drug delivery purposes [127,128]. The advancement of this growing technology also gives the opportunity to combine spatio-temporal designed scaffolds with cells, growth factors, and biomaterials to fabricate multiscale scaffolds that maximally imitate natural tissue characteristics. This process is also widely known as 3D bioprinting [128]. Additive manufacturing also enables the integration of two different material systems in the same scaffold, which ensures better physiological tissue mimicry and enhances the integration of soft and hard tissue, reducing the chances of stress shielding and improving the rate of tissue regeneration [129].

The application of 3D printing with piezoelectric materials was first reported by Kim’s group, who combined BT nanoparticles with PVDF and printed the blend using an FDM system to fabricate a piezoelectric dental implant [17]. Subsequently, after the printing, they also modified the implants using a 1 nm trench laser to fabricate a honeycomb-inspired configuration, which enhances the overall mechanical properties of the implants [15]. Studies have attempted to develop advanced additive manufacturing techniques such as electric poling-assisted additive manufacturing (EPAM) and integrated 3D printing and corona poling (IPC) by combining the corona poling process during 3D printing [129,130]. Such advanced additive manufacturing processes pave the way for rapid scalability of piezo scaffolds by avoiding the post-processing poling stage requirement to enable piezoelectricity [131]. Not only that, after the 3D printing process, one can control the generated electric potential by modulating the amount of mechanical stress, which is also referred to as 4D printing [132]. Collectively, by taking advantage of the 3D printing approach and piezoelectricity, it is possible to fabricate multiscale, multifunctional tissue regeneration strategies.

### 5.4. Other Methods

Apart from the conventional fabrication techniques, piezoelectric materials are also investigated with other formulation systems. For instance, solvent casting, PDMS molding, and spin coating are other common techniques used to fabricate piezoelectric material-based scaffolds [132,133]. For instance, the piezoelectric dermal patch by Bhang et al. [5] was fabricated using layer-by-layer spin coating followed by PDMS curing. Nevertheless, they also reported that a ZnO nanorod-reinforced PDMS patch was also able to generate piezoelectricity during the normal hand rubbing process, which induces the dipole alignment.

## 6. Summary, Conclusions, and Future Perspectives

**Figure 9:**
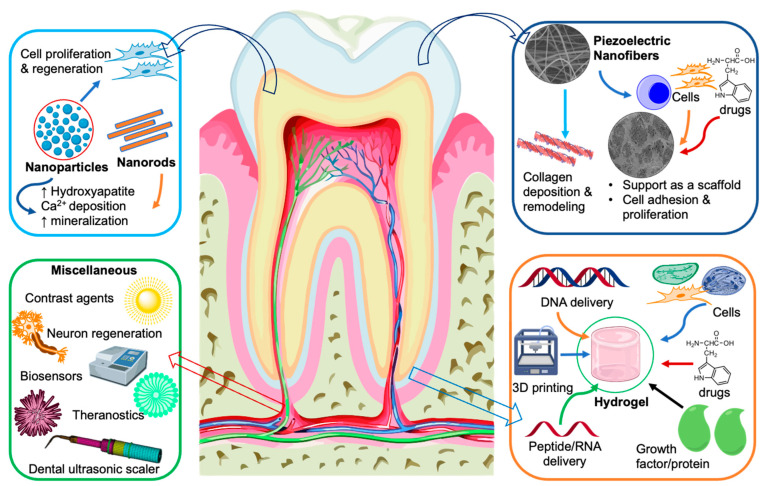
Potential multi-faceted applications of piezoelectric materials in dental tissue engineering.

The highly tunable physicochemical properties and multifunctionality of piezoelectric materials are strong advantages when considering this class of material as scaffolds for dental tissue engineering. Despite the evidence that dental tissues such as dentin have inherent piezoelectric properties and the emerging evidence indicating the advantages of piezoelectric biomaterials, research and development in piezoelectricity-based dental tissue regeneration is extremely limited. Even the studies that reported promising activity for biomaterials did not provide experimental proof to establish and apply piezoelectric behavior for this application. Regarding this aspect, Figure 9 provides a bird’s eye view of the potential applications of smart piezoelectric materials in dentistry. For instance, piezoelectric materials could be fabricated as nanoparticles or nanorods to form antimicrobial or mineralizing agents or, on the other hand, forms such as nanofibers or 3D-printed scaffolds can be explored for guided tissue regeneration purposes. Nevertheless, combination with small therapeutic agents, peptides, or DNA piezo scaffolds could also be investigated as a next-generation drug delivery platform.

However, one major challenge in the clinical translation of piezoelectric biomaterial-based scaffold systems is the validation of whether the stress/mechanical load produced during physiological movement is sufficient to generate piezoelectric potential for the specific biological action. For instance, it has been evidenced that a higher electric potential is preferable for mammalian cell adhesion, proliferation, or tissue regeneration and mineralization purposes whereas a lower electric potential is preferable for bacterial killing. For instance, the best antimicrobial effect was shown by 10% BT-reinforced dental resin composite with an electric potential of 1.2 pC/cm^2^, which contrasts with the maximum mineralization efficiency exhibited by 60% BT-composites with a higher electric potential, i.e., 3.2 pC/cm^2^ [44]. An important research gap in this area is the characterization of whether the same electric potential can kill Gram-positive bacteria, Gram-negative bacteria, and yeasts and eradicate or inhibit biofilms. Another research gap is the lack of a clear elucidation of the mechanisms and molecular signaling pathways by which these materials elicit biological effects both in microbiota and human cells. The long-term biocompatibility of most piezoelectric biomaterials remains to be thoroughly investigated. Furthermore, to develop smart biocompatible piezoelectric scaffold systems, tuning of the piezoelectric characteristics of pristine piezoelectric materials through chemical modification or biomolecule conjugation is an important avenue for future research. 

## 7. Conclusions

The repair and regeneration of the dentin-pulp complex remain a formidable challenge given the sheer biological complexity of this system. The discovery of piezoelectric materials may be considered an important landmark owing to its multifaceted functionalities and excellent tunability. In this review, we discussed how piezoelectric responses generated from piezoelectric biomaterials have been exploited for tissue engineering, antimicrobial, and anti-inflammatory functions. Based on the current review, while it appears plausible that piezoelectric materials may position themselves comfortably as multi-functional biomaterials, the lack of evidence with several outcome measures is also apparent. Despite such voids in the evidence base, these biomaterials may open new doors for successful next-generation regenerative strategies in a field where there is a dire clinical need for innovation and application.

## Figures and Tables

**Table 2:** Piezoelectric biomaterials and their potential applications.

Piezoelectric Biomaterials	Scaffolds	Piezoelectric Coefficients (d_33_)	Potential Applications in Dental Tissue Engineering
Barium Titanate	Composites Dental cement Nanoparticles	191 pC/N [33]	Cell adhesion and proliferation Stem cell differentiation Dental cement Medical imaging Antimicrobial Remineralization
Zinc oxide	Dental cement Nanorods Nanoparticles	3.62 pC/N [34]	Antimicrobial agent Drug delivery Stem cell differentiation Dental cement Medical imaging
PLLA	Composites Hydrogel Nanoparticles	−10 pC/N [35]	Cell adhesion and proliferation Stem cell differentiation Angiogenesis Drug delivery
PHBV	Nanofibers Composites Nanoparticles	1.3 pC/N [36]	Cell adhesion and proliferation Drug delivery
Collagen	Nanofibers 3D-bioprinted scaffold Sponge Nanogel Nanoparticles	0.3 pC/N [36]	Cell adhesion and proliferation Stem cell differentiation Angiogenesis Drug delivery Tissue regeneration Pulp-dentin repair/regeneration
Cellulose	Nanofibers 3D-printed scaffold Sponge Nanogel	0.2–0.4 pC/N [37]	Cell adhesion and proliferation Stem cell differentiation Angiogenesis Drug delivery
Chitosan	Nanofibers 3D-printed scaffold Sponge Nanogel Nanoparticles	0.2–2.0 pC/N [38]	Cell adhesion and proliferation Stem cell differentiation Angiogenesis Drug delivery Tissue regeneration Pulp-dentin repair/regeneration
Silk fibroin	Fibers Porous scaffold	d_14_= −1.5 pC/N [39]	Cell adhesion and proliferation Drug delivery Tissue healing and regeneration

**Table 3:** Reported piezoelectric coefficient value of amino acids/peptide-based piezoelectric biomaterials, measured using PFM or DFT-calculation. The subscript in the brackets indicate the respective directions.

Amino Acid	Piezoelectric Coefficient (pC/N)	Reference
Threonine	4.9 pC/N (d_36_)	[111]
Proline	27.75 pC/N (d_25_)	[111]
Asparagine	13 pC/N (d_16_)	[111]
Histidine	18 pC/N (d_16_)	[111]
Leucine	12.5 pC/N (d_16_)	[112]
Isoleucine	25 pC/N (d_34_)	[111]
Cysteine	11.4 pC/N (d_22_)	[111]
Glycine	178 pC/N (d_16_)	[113]
Alanine	17.75 pC/N (d_24_)	[113]
**Poly-Amino Acid/Peptide**	**Piezoelectric Coefficient (pC/N)**	**Reference**
Keratin	1.8 pC/N (d_14_)	[106]
Lysozyme	6.5 pC/N (d_33_)	[106]
Diphenylalanine (FF)	80 pC/N (d_15_)	[109]
poly(γ-benzyl-α,L-glutamate) PBLG	25 pC/N (d_33_)	[114]
poly-γ-methyl-l-glutamate (PMLG)	2 pC/N (d_14_)	[115]

## Data Availability

No original datasets were generated in this review.
